# miR-155 mediates arsenic trioxide resistance by activating Nrf2 and suppressing apoptosis in lung cancer cells

**DOI:** 10.1038/s41598-017-06061-x

**Published:** 2017-09-22

**Authors:** Shiyan Gu, Yanhao Lai, Hongyu Chen, Yuan Liu, Zunzhen Zhang

**Affiliations:** 1 Department of Environmental Health and Occupational Medicine, West China School of Public Health, Sichuan University, Chengdu, Sichuan 610041 China; 2 Department of Chemistry and Biochemistry, Florida International University, Miami, Florida 33199 USA; 3 Biochemistry Ph.D. Program, Florida International University, Miami, Florida 33199 USA; 4 Biomolecular Sciences Institute, Florida International University, Miami, Florida 33199 USA

## Abstract

Arsenic trioxide (ATO) resistance is a challenging problem in chemotherapy. However, the underlying mechanisms remain to be elucidated. In this study, we identified a high level of expression of miR-155 in a human lung adenocarcinoma A549R cell line that is highly resistant to ATO. We showed that the high level of miR-155 was associated with increased levels of cell survival, colony formation, cell migration and decreased cellular apoptosis, and this was mediated by high levels of Nrf2, NAD(P)H quinone oxidoreductase 1 (NQO1), heme oxygenase-1 (HO-1) and a high ratio of Bcl-2/Bax. Overexpression of the miR-155 mimic in A549R cells resulted in increased levels of colony formation and cell migration as well as reduced apoptosis along with increased Nrf2, NQO1 and HO-1. In contrast, silencing of miR-155 expression with its inhibitor in the cells, significantly decreased the cellular levels of Nrf2, NQO1 and HO-1 as well as the ratio of Bcl-2/Bax. This subsequently reduced the level of colony formation and cell migration facilitating ATO-induced apoptosis. Our results indicate that miR-155 mediated ATO resistance by upregulating the Nrf2 signaling pathway, but downregulating cellular apoptosis in lung cancer cells. Our study provides new insights into miR-155-mediated ATO resistance in lung cancer cells.

## Introduction

Arsenic trioxide (As_2_O_3_, ATO) has been successfully used in the treatment of relapsed/refractory acute promyelocytic leukemia (APL) since 1970s^1^. It is also used as a treatment of solid tumors such as hepatic sarcoma, prostate, and renal cancer among others^2–4^. It has been shown that ATO can induce cancer cell death by causing oxidative stress, DNA damage, and apoptosis^5^. Studies from our group and others have demonstrated that ATO also causes cell death in lung cancer cells^6,7^ indicating that ATO may be employed for lung cancer treatment. However, the doses for ATO to induce lung cancer cell death are much higher than those for the treatment of hematologic malignancies^6–8^, indicating that lung cancer cells are more resistant to ATO than hematologic cancer cells. Since a high dose of ATO can result in severe side effects^9^, this hinders the preclinical trials of ATO for lung cancer treatment. Thus, it is critically important to study the mechanisms underlying ATO resistance of lung cancer cells as this will help identify novel targets for attenuating ATO resistance, thereby facilitating the application of ATO as a new treatment for lung cancer.

One of the important mechanisms that underlie anticancer drug resistance is the high level and capacity of antioxidants in cancer cells^10^, which are primarily regulated by the nuclear factor (erythroid-derived 2)-like 2 (Nrf2) and kelch-like ECH-associated protein-1 (KEAP1) signaling pathway, one of the most important cell defense and survival pathways^11^. Nrf2 is a critical transcription regulator of a series of antioxidants and detoxification enzymes. By uncoupling with KEAP1, Nrf2 initiates the expression of antioxidant genes including NAD(P)H quinone oxidoreductase 1 (NQO1) and heme oxygenase-1 (HO-1)^11,12^. However, previous studies have shown that cancer cells that exhibit a high level of Nrf2 are less sensitive to chemotherapeutic agents^13^. Moreover, an aberrant accumulation of Nrf2 in cancer cells confers cancer resistance to chemotherapeutic agents^13^. Because this can create an environment that promotes cancer cell growth and metastasis, but prevents cancer cells from apoptosis, thereby leading to tumor reoccurrence and poor prognosis in cancer patients^12^. Our previous studies have shown that ATO significantly increases the level of Nrf2 in a human lung carcinoma cell line, A549 cell line^14^, suggesting that upregulation of Nrf2 is involved in resistance of A549 cells to ATO. However, the mechanism underlying Nrf2-mediated cellular resistance to ATO in lung cancer cells remains to be elucidated.

MicroRNAs (miRNAs) are a class of small non-coding RNAs (19-25 nt) that regulate protein translation and stability of mRNA^15^. miRNAs downregulate gene expression by binding to the 3′-untranslated region (3′-UTR) of a target mRNA, thereby inducing degradation of mRNAs and silencing the expression of a target gene^15^. It has been found that miRNAs play critical roles in many biological processes including cell proliferation and survival^15^. Dysregulation of miRNAs modulates the initiation and progression of cancer^16^. Moreover, a growing body of evidence indicates that several miRNAs may mediate cellular resistance to chemotherapy and radiotherapy in various types of tumors and cancer, in particular, lung cancer^17^. Among all of the identified miRNAs, miR-155 is the one that has been characterized extensively. miR-155 is generated from an exon of a non-coding RNA known as B-cell Integration Cluster (BIC)^18^. It is involved in cancer initiation and progression as well as the development of cellular resistance to chemotherapeutic agents^17,19–21^. A previous study has shown that the level of miR-155 in lung cancer tissue is much higher than that in normal tissue^22^. Moreover, lung adenocarcinoma patients who exhibited a high level of miR-155 in the cancer tissue usually had poor prognosis^20,22^. Inhibition of miR-155 expression suppressed cancer cell proliferation and promoted apoptosis, thereby sensitizing cancer cells to chemotherapeutic agents, cisplatin and doxorubicin^19,21^. Interestingly, it has been also shown that miR-155 can upregulate NQO1 and HO-1 through activation of the Nrf2 signaling pathway, thereby protecting cells against oxidative stress^23,24^. This further indicates that miR-155 can modulate cell proliferation and apoptosis via regulation of cell redox homeostasis. Our previous results have shown that a high dose of ATO can reduce the total antioxidant capacity of lung cancer cell (A549) by inhibiting cellular expression of miR-155, leading to cancer cell death (unpublished data). However, the mechanisms by which A549 cells develop ATO resistance through miR-155 remains unknown. To address this, we established a lung cancer cell line that is highly resistant to ATO, the A549R cell line, as a model to study the mechanisms by which miR-155 mediates ATO resistance in lung cancer cells. We discovered that a high level of miR-155 and activation of the Nrf2 signaling pathway along with decreased cellular apoptosis led to ATO resistance of A549R. This was further confirmed by the fact that overexpression of the miR-155 mimic enhanced ATO resistance of A549 via increased levels of NQO1 and HO-1 as well as an increased ratio of Bcl-2/Bax, whereas silencing of miR-155 with its inhibitor significantly increased the cellular sensitivity to ATO, and this resulted from decreased levels of NQO1 and HO-1 proteins as well as an decreased ratio of Bcl-2/Bax. The results indicated that miR-155 mediated cellular ATO resistance by activating the Nrf2 signaling pathway and suppressing apoptosis. Our study provided new insights into the mechanisms underlying ATO resistance in lung cancer cells. We suggest that miR-155 can be developed as a new therapeutic target for combating ATO resistance in lung cancer.

## Results

### The ATO resistance of A549R cells

**Figure 1:**
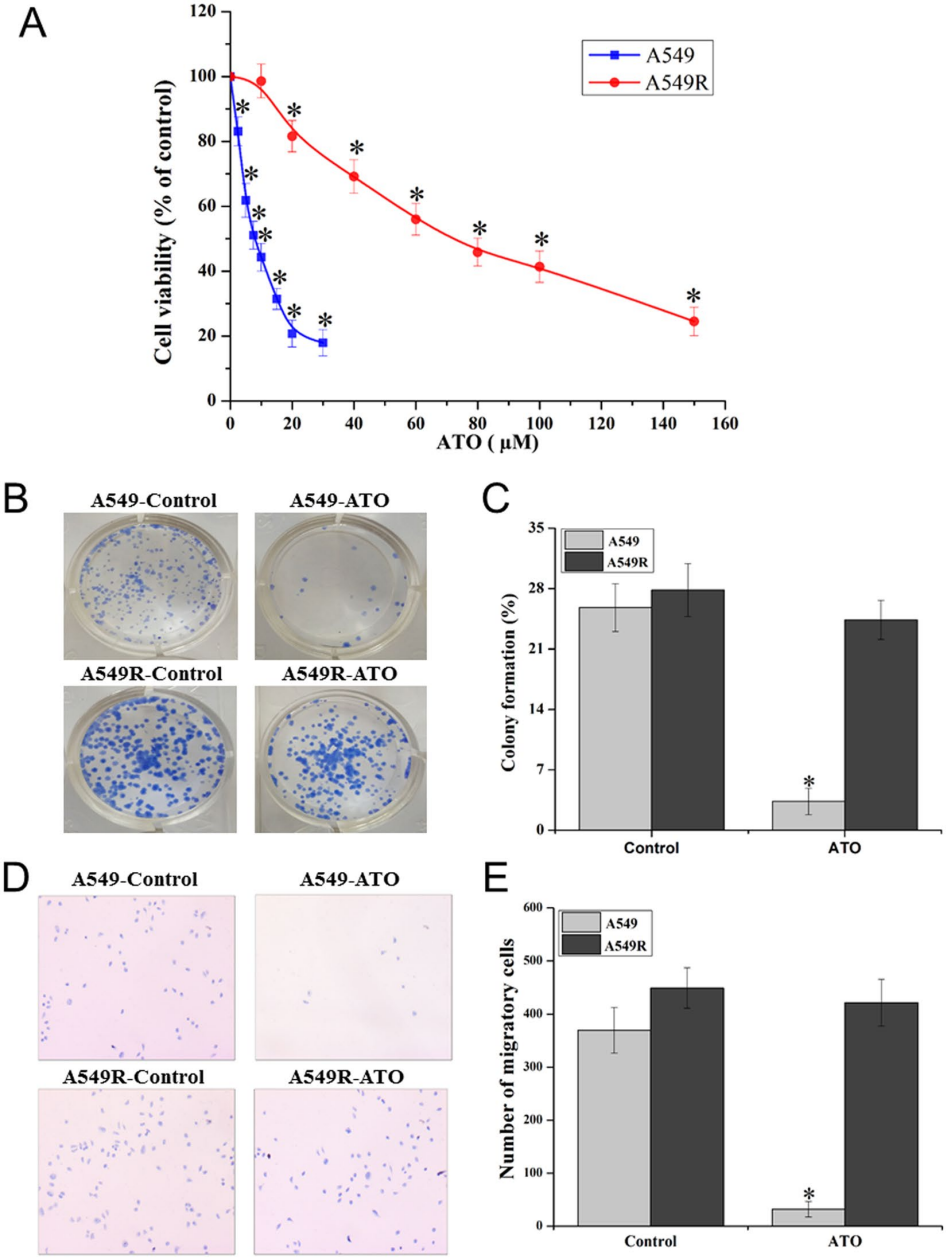
ATO resistant phenotypes in A549 and A549R cells. ATO resistant phenotypes were determined by MTT assay, colony formation, and cell migration assays. (**A**) The viability of A549 and A549R cells treated with different concentrations of ATO ranging from 0 to 30 µM and 0 to 150 µM for 72 hours. (**B**,**C**) The colony formation images and the percentage of colony formation of A549 and A549R cells that were treated without or with 20 µM ATO for 24 hours. (**D**,**E**) The microscopic images of migratory cells (100×) and the number of migrating cells of A549 and A549R cells without or with ATO treatment. Results were obtained from three independent experiments and presented as mean ± standard deviation (SD). *P* < 0.05 was designated as statistical significance. “*” indicates a significant difference compared with the corresponding control groups (*P* < 0.05). “#” indicates a significant difference compared with A549 cells (*P* < 0.05).

To determine if A549R cells exhibit a high level of resistance to ATO, we initially measured the viability of A549R cells under treatment of different doses of ATO in comparison to that of A549 cells with MTT assay. We found that the viability of A549 cells was significantly decreased by ATO that ranged from 2.5 µM to 30 µM (Fig. 1A). Treatment with 10 µM ATO for 72 hours, led to the death of more than 50% of A549 cells, whereas treatment with 20 µM–30 µM ATO resulted in the death of 80% of A549 cells (Fig. 1A). In contrast, under the same conditions, 10 µM ATO did not exhibit any cytotoxic effects on A549R cells, and 20 µM ATO only led to 20% cell death (Fig. 1A). The viability of A549R cells decreased along with increasing concentrations of ATO above 20 µM. The concentrations of ATO that caused the death of half of A549R cells fell into the range of 60 µM–80 µM, which were about 10-fold higher than that of A549 cells. The 50% inhibitory concentrations (IC_50_) of ATO for A549 and A549R cells were 8.02 µM and 76.81 µM, respectively. Based on the IC_50_, the drug resistance index of A549R cells was calculated as 9.58. The results indicated that A549R cells exhibited a significantly high level of resistance to ATO. To further verify the high level of resistance of A549R cells to ATO, we determined the colony formation of A549R and A549 cells without or with treatment of 20 µM ATO for 24 hours (Fig. 1B and C). Without treatment of ATO, both A549 and A549R cells exhibited a similar percentage of colony formation with 25.80% from A549 cells and 27.83% from A549R cells, respectively (Fig. 1B and C). With the treatment of 20 µM ATO, A549 cells exhibited only 3.33% of colony formation, whereas A549R cells exhibited 24.37% of colony formation (*P* < 0.05) (Fig. 1B and C). The results confirmed that A549R cells exhibited much higher resistance to ATO than A549 cells. We then examined the migration of A549R cells under ATO treatment to verify the drug resistant phenotype with a two-chamber system composed of an upper and lower chamber. Cell migration was measured by counting the number of cells which migrated from the upper chamber to the lower chamber (Fig. 1D and E). As shown in Fig. 1D and E, without ATO treatment, a similar number of A549 (361) and A549R cells (430) migrated from the upper chamber into lower chamber indicating that they have the similar ability of migration and survival. However, under treatment of 20 µM ATO, only 33 A549 cells migrated, whereas 421 A549R cells migrated. This indicates that A549R cells readily survived to migrate under the ATO treatment (Fig. 1E and F). Thus, the results demonstrated that A549R cells were much more resistant to ATO than A549 cells.

### A549R cells exhibit poor apoptotic activity under ATO treatment

**Figure 2:**
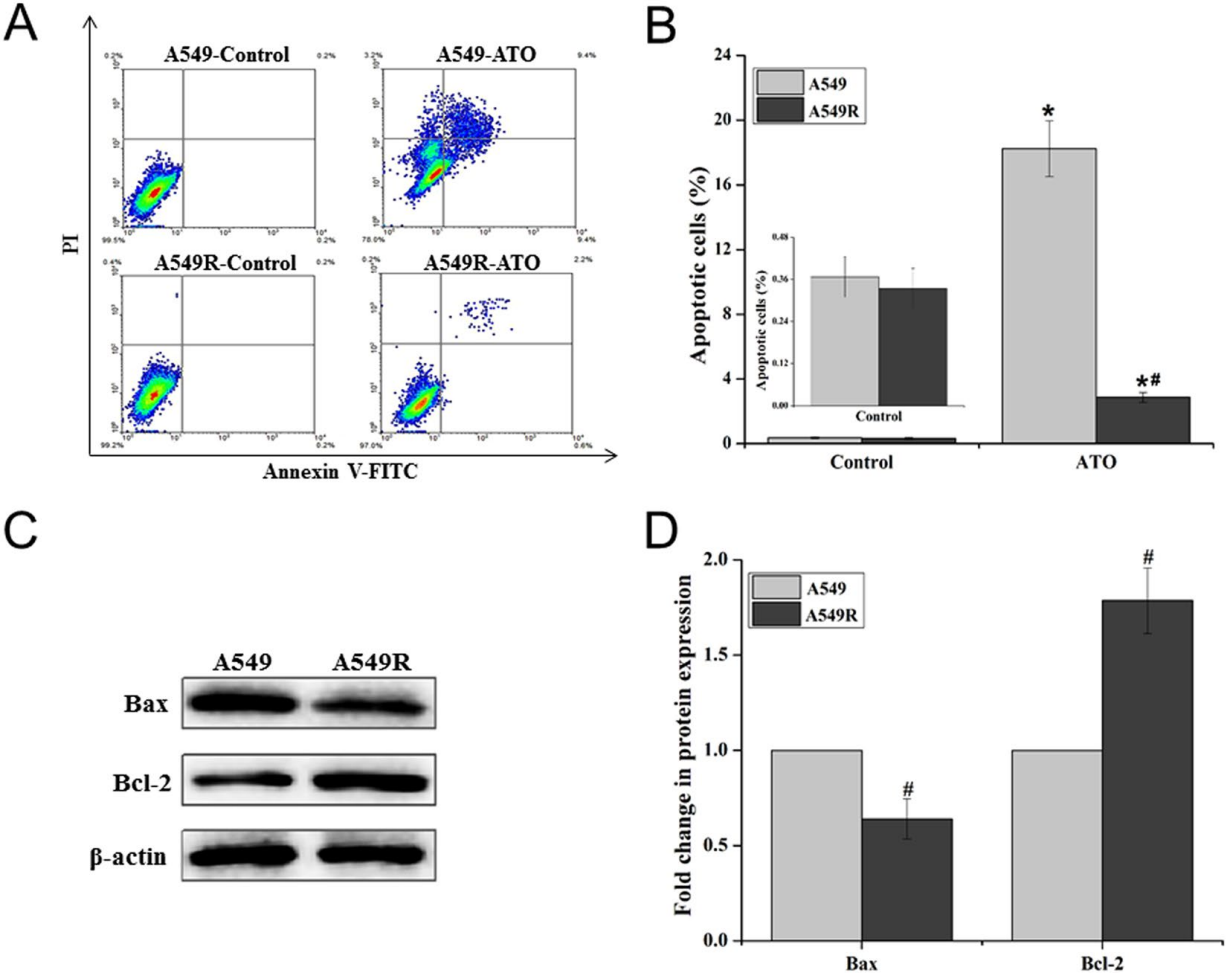
ATO-induced apoptosis in A549 and A549R cells. A549 and A549R cells were treated without or with 20 µM ATO for 24 hours. Apoptotic cells were detected by Annexin V-FITC/PI dual staining using flow cytometry, and the percentage of apoptotic cells was calculated. Cellular levels of apoptosis-associated protein Bax and Bcl-2 were detected by immunoblotting. (**A**) The flow cytometry profiles of apoptotic cells in A549 and A549R cells without or with ATO treatment. (**B**) The percentage of apoptotic cells in A549 and A549R cells under different treatments. (**C**,**D**) The expression of Bax and Bcl-2 proteins in A549 and A549R cells and “Fold change” of the cellular level of Bax and Bcl-2 proteins. The blots were cropped for the clarity of the results. Results were obtained from three independent experiments, and quantitative results are presented as mean ± standard deviation (SD). *P* < 0.05 was designated as statistical significance. “*” indicates a significant difference compared with corresponding control group (*P* < 0.05). “#” indicates a significant difference compared with A549 cells (*P* < 0.05).

Since apoptosis can mediate ATO-induced cell death, we asked whether the high level of resistance of A549R cells to ATO was due to the modulation of apoptotic process. To address this question, we determined the percentage of apoptotic cells in untreated A549 and A549R cells as well as in the cells treated with 20 µM ATO for 24 hours and the level of apoptotic proteins in the cells. We found that lower than 0.36% of apoptotic cells were detected in untreated A549 and A549R cells (Fig. 2A, left panels and Fig. 2B). However, 20 μM ATO increased the percentage of apoptotic cells in A549 and A549R cells to 18% and 3%, respectively (Fig. 2A, right panels and Fig. 2B). Thus, A549R cells exhibited 6-fold lower percentage of apoptotic cells than A549 cells (Fig. 2A and B). This indicated that A549R cells exhibited much lower apoptotic activity than A549 cells under treatment of ATO. To further identify the molecular basis underlying the phenomena, we determined the expression levels of the pro-apoptotic protein Bax and anti-apoptotic protein Bcl-2 in the cells. We found that the level of Bax in A549R cells was lower than that in A549 cells (Fig. 2C and D), whereas the level of Bcl-2 in A549R cells was higher than that in A549 cells (Fig. 2C and D). This resulted in a lower ratio of Bax/Bcl-2 in A549R cells.

### Nrf2 signaling pathway contributes to ATO resistance of A549R cells

**Figure 3:**
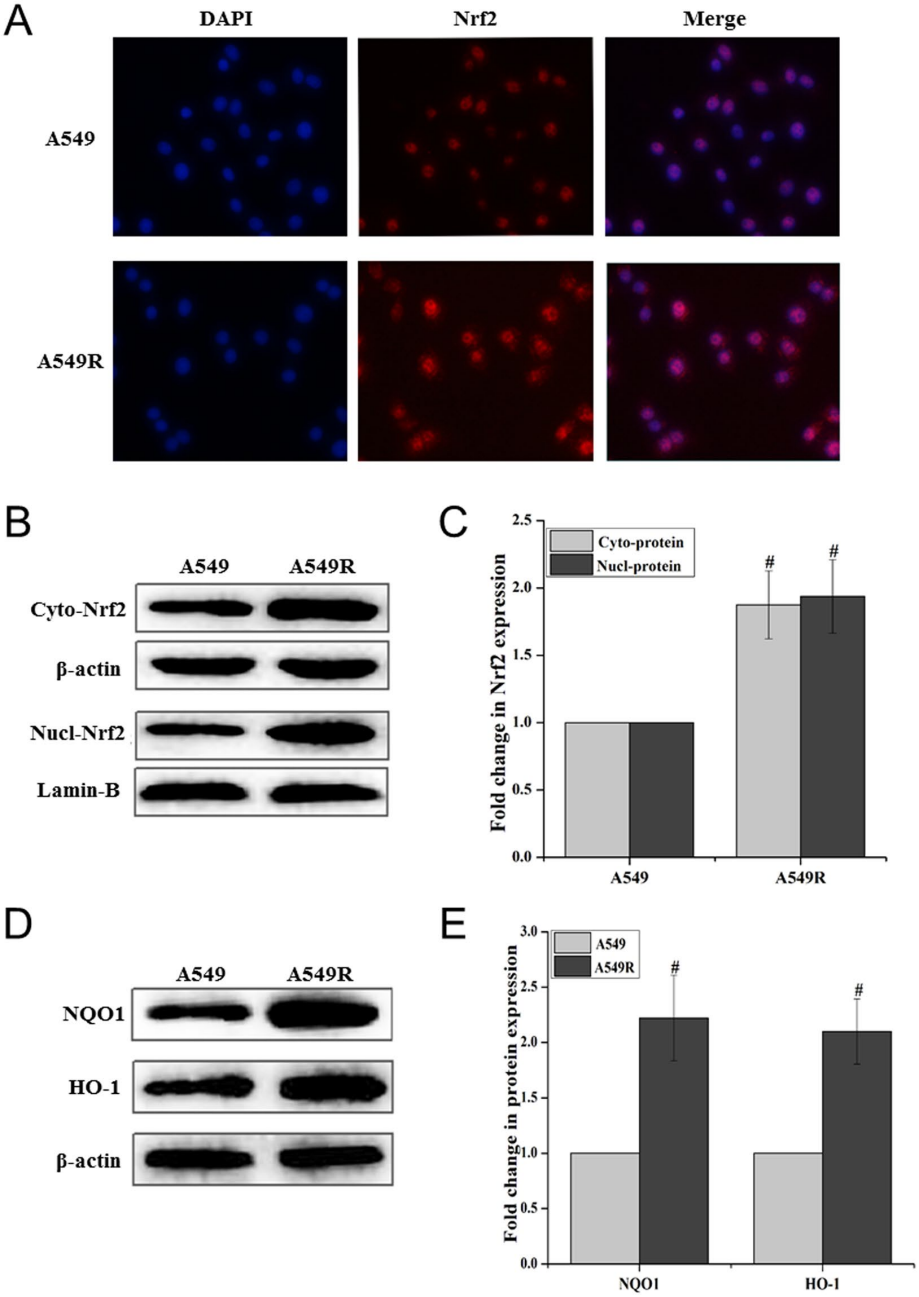
Activation of the Nrf2 signaling pathway in A549 and A549R cells. The cellular location and expression of the Nrf2 signaling pathway proteins in A549 and A549R cells were detected with immunofluorescence and immunoblotting. (**A**) Nrf2 nuclear and cytoplasmic localization (400×) was detected with immunofluorescence. (**B**,**C**) The expression of Nrf2 in the cytoplasm and nucleus of A549 and A549R cells. The “Fold change in Nrf2 expression” was calculated based on the level of Nrf2 in A549 cells. (**D**,**E**) The “Fold change in protein expression” of NQO1, HO-1 in A549R cells was calculated based on the levels of the proteins in A549 cells. The blots were cropped for the clarity of the results. Results were obtained from three independent experiments, and quantitative results are presented as mean ± standard deviation (SD). “#” indicates a significant difference compared with A549 cells (*P* < 0.05).

Nrf2 mainly upregulates the expression of cellular antioxidant genes such as NQO1 and HO-1^12,13^. Since our previous studies have shown that activation of Nrf2 is involved in ATO resistance in A549 cells^14^, it is conceivable that the Nrf2 signaling pathway may also mediate the high level of ATO resistance in A549R cells by increasing the cellular antioxidant capacity of the cells. To test this, we examined the distribution of Nrf2 in the nucleus and cytoplasm with immunofluorescence. We found that Nrf2 existed in both the nucleus and cytoplasm of A549 cells and A549R cells (Fig. 3A). However, the level of Nrf2 protein in the nucleus and cytoplasm of A549R cells was significantly higher than that in A549 cells (Fig. 3B and C). Accordingly, the levels of antioxidant proteins, HO-1 and NQO1 in A549R cells were also much higher than those in A549 cells (Fig. 3D and E). The results indicate that the Nrf2 signaling pathway was more active in A549R cells than in A549 cells.

### miR-155 promotes the resistance of A549R cells to ATO

**Figure 4:**
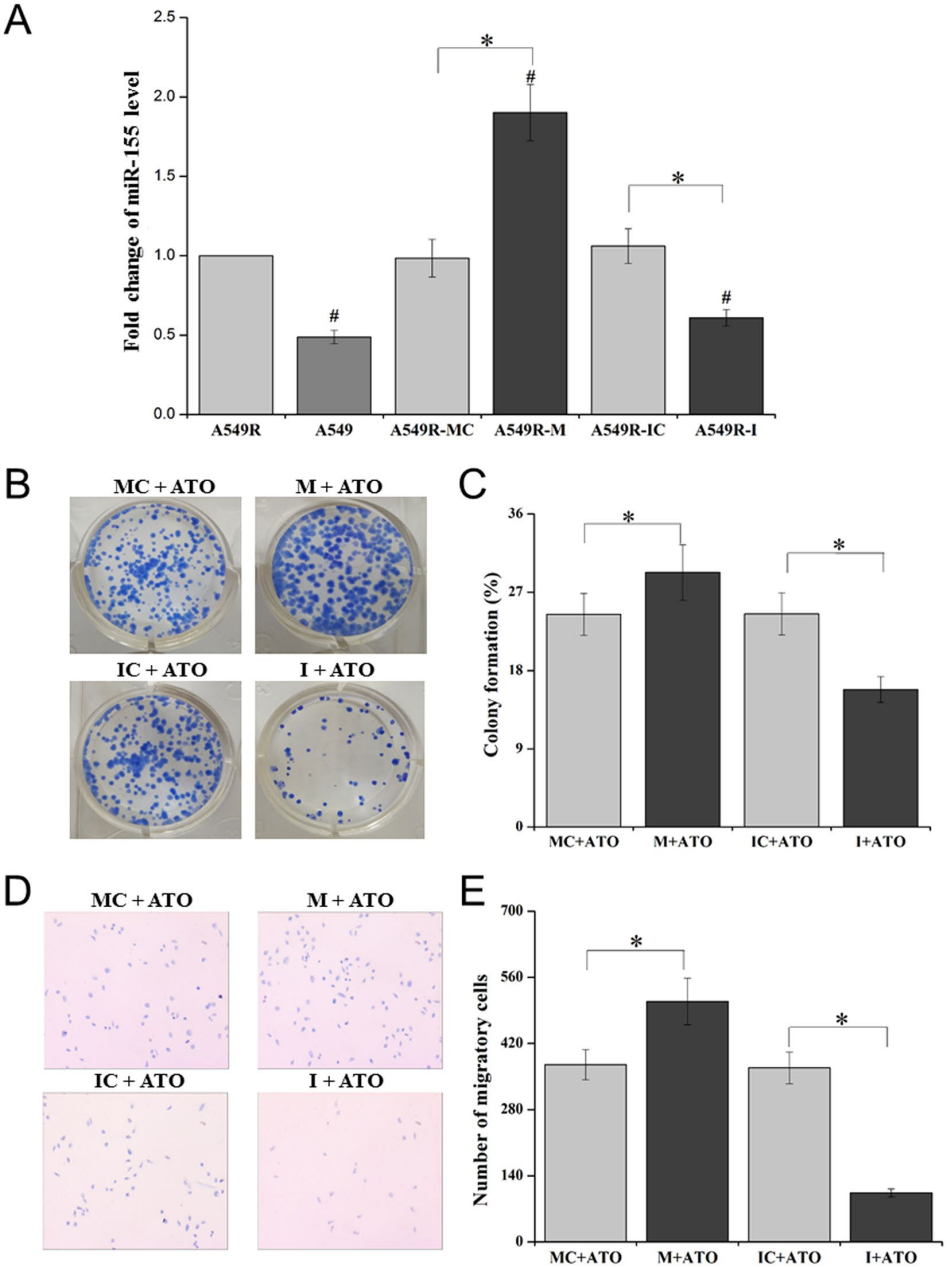
The level of miR-155 in A549 and A549R cells. The level of miR-155 in A549 and A549R cells as well as A549R cells transfected with the miR-155 mimic control (**MC**), miR-155 mimic (**M**), miR-155 inhibitor control (**IC**) and miR-155 inhibitor (**I**) was determined with real-time PCR. (**A**) The miR-155 level in A549, A549R, and A549R cells transfected with **MC**, **M**, **IC** and **I**. The “Fold change in the level of mi-R155” was calculated based on the miR-155 level in A549R cells. The results of cell colonies and the percentage of colony formation were illustrated in (**B**,**C**), respectively. (**D**,**E**) The image of migratory cells (100×) and the number of migrating cells of A549R cells. Results were obtained from three independent experiments, and the quantitative results are presented as mean ± standard deviation (SD). “*” indicates a significant difference compared with the cells transfected with **MC** and **IC** (*P* < 0.05).

miR-155 expression is associated with increased cell proliferation and chemotherapeutic drug resistance such as cellular resistance to cisplatin and doxorubicin^19,21^. Recent studies have also shown that miR-155 can upregulate the expression of NQO1 and HO-1 gene to increase cellular antioxidative capacity and cell survival^23,24^. Thus, it is possible that miR-155 may mediate ATO resistance in A549R cells by upregulating NQO1 and HO-1 gene expression. To test this, we measured the level of miR-155 in A549 and A549R cells and found that the level of miR-155 in A549R cells was significantly higher than that in A549 cells (Fig. 4A). Moreover, introduction of the miRNA-155 mimic (M) or the miRNA-155 inhibitor (I) into A549R cells increased or decreased the level of miR-155 by about 2-fold compared with that of the miR-155 mimic control (MC) or the miR-155 inhibitor control (IC) (Fig. 4A). The results indicate that the miR-155 mimic or the miR-155 inhibitor can be employed to effectively regulate the level of miR-155 in A549R cells. Further characterization of the effects of miR-155 on the growth and proliferation of A549R cells under ATO treatment showed that A549R cells transfected with the miR-155 mimic exhibited a higher percentage of colony formation (29.27%) than the control (24.43%) (*P* < 0.05) (Fig. 4B and C), whereas A549R cells transfected with the miR-155 inhibitor exhibited a lower percentage of colony formation (15.83%) than the control (24.50%) (*P* < 0.05) (Fig. 4B and C). Similarly, under treatment of 20 µM ATO, 509 A549R cells transfected with the miR-155 mimic, migrated, whereas 375 A549R cells transfected with the control vector, migrated indicating that upregulation of miR-155 promoted the migration and survival of A549R cells (Fig. 4D and E). However, under the same treatment, only a small number of A549R cells transfected with the miR-155 inhibitor, migrated (Fig. 4D and E) indicating that downregulation of miR-155 suppressed cell migration and survival. The results indicate that overexpression of miR-155 promoted A549R cell survival and cellular resistance to ATO, whereas suppression of miR-155 expression inhibited A549R survival and reduced cellular resistance to ATO. This further indicated that miR-155 mediated ATO resistance of A549R cells.

### miR-155 downregulates ATO-induced apoptosis in A549R cells

**Figure 5:**
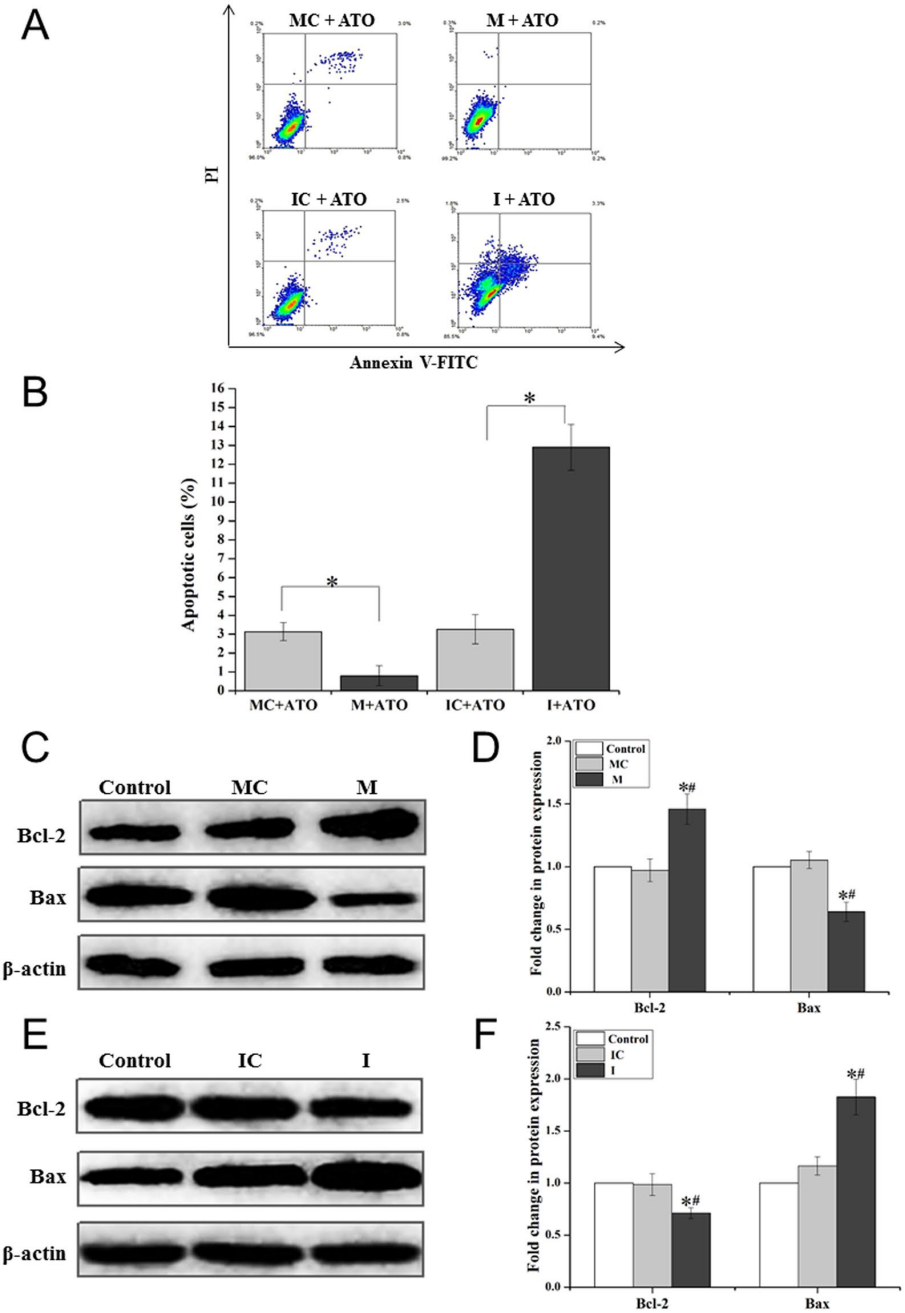
miR-155 attenuates ATO-induced apoptosis in A549R cells. A549R cells were transfected with the miR-155 mimic negative control (**MC**), the miR-155 mimic (**M**), the miR-155 inhibitor negative control (**IC**) and miR-155 inhibitor (**I**). Annexin V-FITC/PI dual staining assay was used to quantify the percentage of apoptotic cells in A549R cells that were treated without or with 20 µM ATO for 24 hours. Cellular level of anti-apoptotic protein Bcl-2 and pro-apoptotic protein Bax were determined by immunoblotting. (**A**) The profiles of flow cytometry for determining apoptosis in A549R cells. (**B**) The percentage of apoptotic cells in different experimental groups. Representative immunoblotting images of Bax and Bcl-2 proteins in untransfected cells (Control) and cells transfected with **MC**, **M**, **IC** and **I** were shown in (**C**,**E**). The level of β-actin was used as a loading control. The “Fold change in protein expression” of Bax and Bcl-2 was calculated based on the level of protein expression in the untransfected A549R control cells in (**E**,**F**). The blots were cropped for the clarity of the results. Results were obtained from three independent experiments, and quantitative results were presented as mean ± standard deviation (SD). “*” indicates a significant difference compared with a negative control group (*P* < 0.05). “#” indicates a significant difference compared with untransfected A549R cells (Control) (*P* < 0.05).

Since we found that ATO-induced apoptosis was significantly reduced in A549R cells (Fig. 3), it is possible that apoptosis in A549R cells may be downregulated by miR-155. To test this possibility, we determined the percentage of apoptotic cells in A549R cells that were transfected with the miR-155 mimic or the miR-155 inhibitor under treatment of 20 µM ATO for 24 hours (Fig. 5A and B). The results showed that ATO resulted in a low percentage of apoptotic cells (0.8%) in A549R cells transfected by the miR-155 mimic, and a high percentage of apoptotic cells (12.9%) in the cells transfected with the miR-155 inhibitor (Fig. 5A and B, right panels). However, ATO treatment only resulted in 3.27% and 3.13% apoptotic cells in A549R cells transfected with the vector controls of the miR-155 mimic and the miR-155 inhibitor, respectively (Fig. 5A and B, left panels). This indicated that miR-155 efficiently downregulated apoptosis. Since a low ratio of Bax/Bcl-2 can mediate anti-cancer drug resistance by preventing the initiation of apoptosis^25^, we then asked if up- or downregulation of miR-155 could up- or downregulate apoptosis by modulating the ratio of Bax/Bcl-2. To address this, we examined the expression level of Bax and Bcl-2 proteins in A549R cells transfected with the miR-155 mimic or the miR-155 inhibitor. We found that in A549R cells transfected with the miR-155 mimic, the level of Bcl-2 and Bax was 1.46- and 0.64-fold of those in untransfected cells, respectively (Fig. 5C and D). However, the levels of Bcl-2 and Bax in A549 cells transfected with the miR-155 inhibitor was 0.71- and 1.83-fold of those in untransfected cells (Fig. 5E and F). Thus, the miR-155 mimic decreased the ratio of Bax/Bcl-2 in A549R cells to 0.44, whereas the miR-155 inhibitor elevated the Bax/Bcl-2 ratio to 2.58. The results indicate that miR-155 suppressed the initial response of A549R cells to apoptosis by decreasing the ratio of Bax/Bcl-2 through upregulating Bcl-2 and downregulating Bax.

### Effects of miR-155 on the expression of Nrf2, HO-1 and NQO1 in A549R cells

**Figure 6:**
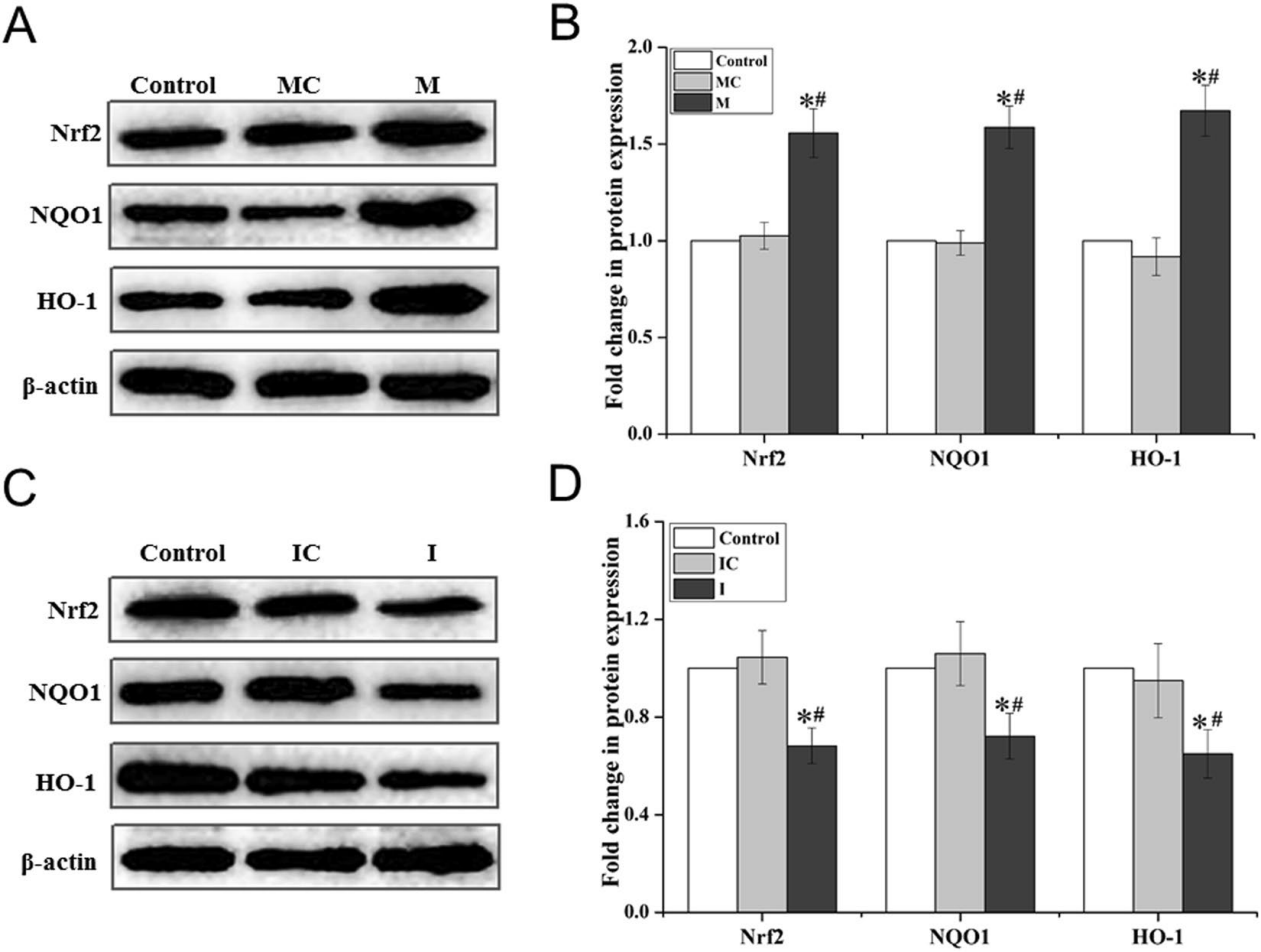
The effects of miR-155 on the level of Nrf2, HO-1 and NQO1 proteins in A549R cells. A549R cells were transfected with the miR-155 mimic negative control (**MC**), the miR-155 mimic (**M**), the miR-155 inhibitor negative control (**IC**) and imR-155 inhibitor (**I**). The protein levels of Nrf2, HO-1 and NQO1 were determined with immunoblotting. (**A**,**C**) showed representative immunoblots. (**B**,**D**) The “Fold change in protein expression” of Nrf2, HO-1 and NQO1, which was calculated based on the protein level in the untransfected A549R control cells. The blots were cropped for the clarity of the results. Results were obtained from three independent experiments, and quantitative results are presented as mean ± standard deviation (SD). “*” indicates a significant difference compared with the negative control groups (**MC** or **IC**)(*P* < 0.05). “#” indicates a significant difference compared with untransfected A549R cells (Control) (*P* < 0.05).

Previous studies have shown that the expression of cellular antioxidants, HO-1 and NQO1can be regulated by miR-155^23,24^. We have also discovered that HO-1 and NQO1 are involved in cellular ATO drug resistance^26^. To further determine if miR-155 can mediate the resistance of A549R cells to ATO by regulating cellular levels of Nrf2, HO-1 and NQO1, we examined the levels of Nrf2, HO-1 and NQO1 in A549R cells transfected with the miR-155 mimic, the miR-155 inhibitor, the miR-155 mimic control and miR-155 inhibitor vector control, respectively. We found that the levels of Nrf2, HO-1 and NQO1 proteins were increased by about 1.5-fold in A549R cells transfected with the miR-155 mimic compared with those in the cells transfected with the mimic vector control and untransfected A549R cells (*P* < 0.05) (Fig. 6A and B). In contrast, the levels of HO-1 and NQO1 proteins were decreased by about 0.6-fold in A549R cells transfected with the miR-155 inhibitor compared with those in cells transfected with the inhibitor vector control (Fig. 6C and D). The results indicate that miR-155 activated the Nrf2 signaling pathway to increase the levels and capacity of antioxidants in A549R cells. This in turn enhanced the resistance of A549R cells to ATO.

## Discussions

**Figure 7:**
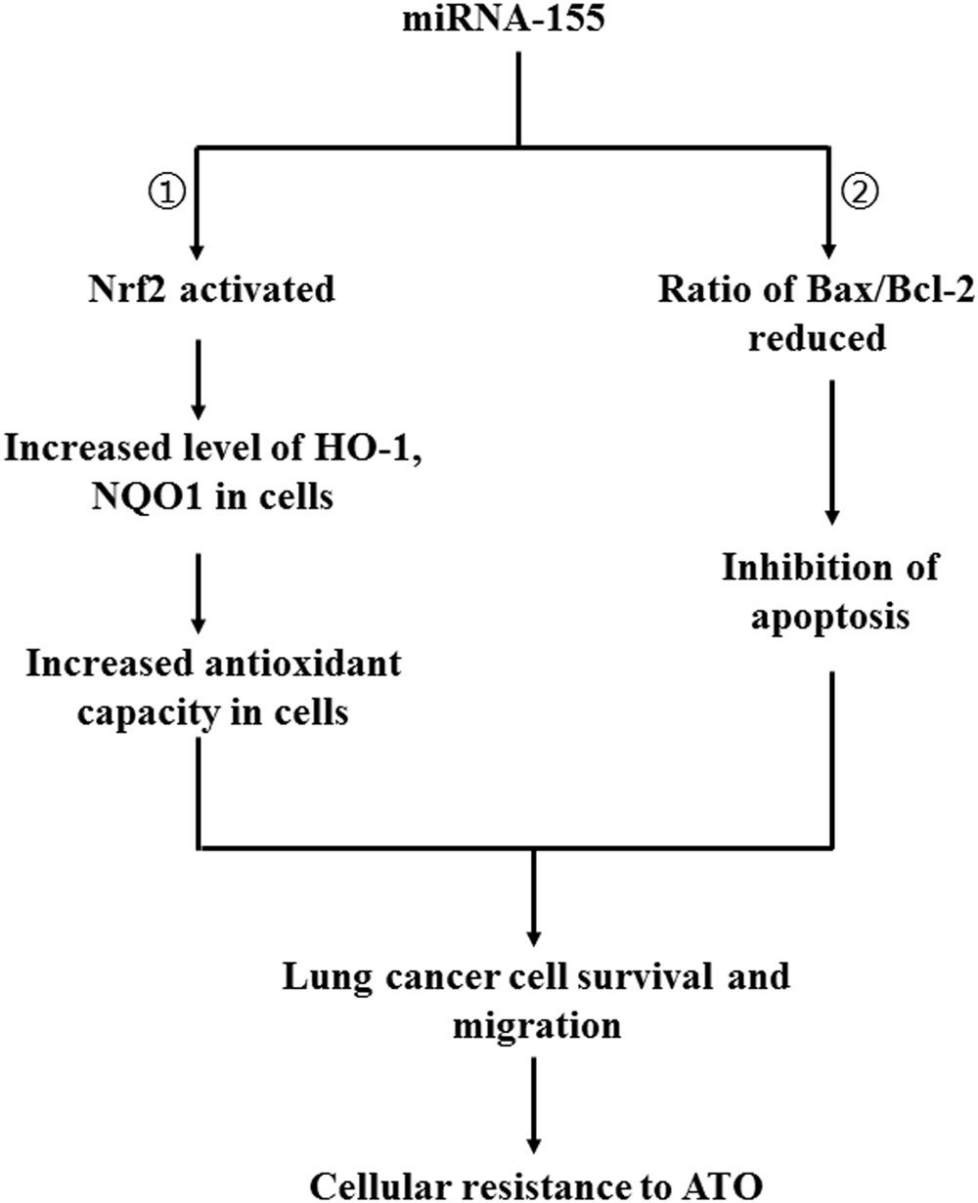
The pathways for miRNA-155 to mediate ATO resistance in A549R cells. miR-155 in lung cancer cells activates Nrf2 that subsequently upregulates the expression of antioxidant genes i.e., HO-1 and NQO1 in A549R cells (subpathway 1). This further increases the antioxidant capacity and promotes survival of the lung cancer cells. On the other hand, miR-155 reduces the ratio of Bax/Bcl-2 and suppressed apoptosis and cell death (subpathway 2). All these result in increased colony formation and migration, thereby leading to a high level of cellular resistance to ATO.

In this study, we initially characterized a lung adenocarcinoma cell line, i.e. A549R cells that exhibited a significantly high level of resistance to ATO than A549 cells (Figs 1 and 2). We then explored the mechanisms underlying the ATO-resistance of A549R cells. We provided the first evidence that miR-155 mediated a high level of ATO-resistance in a lung cancer cell line, A549R cells by suppressing cellular apoptotic activities and upregulating the expression of antioxidants NQO1 and HO-1 via activation of the Nrf2 signaling pathway (Figs 2–6), and this subsequently protected A549R cells from ATO-induced apoptotic cell death, thereby promoting cell survival (Figs 4 and 5). The results support a model that miR-155 mediates the resistance of A549R cells to ATO through a pathway in which miR-155 increases the level of Nrf2 in the nucleus and cytoplasm of A549R cells. This subsequently upregulates the expression of antioxidant proteins NQO1 and HO-1 that protect cells from ATO-induced oxidative stress. On the other hand, miR-155 upregulates Bcl-2 and downregulates Bax, resulting in a high ratio of Bcl-2/Bax that subsequently prevents apoptosis. All of these promote cell survival and a high level of cellular resistance to ATO (Fig. 7).

miR-155 is implicated in the initiation and progression of cancer as well as the development of drug resistance in cancer cells^22,23^. It has been shown that silencing of miR-155 leads to overexpression of the pro-apoptotic protein, Apaf-1, which increases the sensitivity of A549 cells to cisplatin^21^. Similarly, suppression of miR-155 in doxorubicin-resistant A549 cells (A549/dox) can significantly reduce doxorubicin resistance^19^. The results indicate that miR-155 plays a universal role in mediating anticancer drug resistance in A549 cells. In this study, we further demonstrated that miR-155 was also involved in mediating the resistance of A549R cells to ATO. Since miR-155 can upregulate NQO1 and HO-1 through activation of the Nrf2 signaling pathway to promote cellular redox homeostasis and cell survival^23,24^, it appears that miR-155 may also mediate the resistance of A549R lung cancer cells to ATO by activating the Nrf2 signaling pathway and its downstream target genes. Indeed, this notion was supported by our results showing that the expression of Nrf2, NQO1 and HO-1 was upregulated along with upregulation of miR-155 in A549R cells (Figs 3 and 4A). This was further supported by the fact that overexpression of miR-155 in A549R cells increased the levels of Nrf2, NQO1 and HO-1, whereas suppression of the expression of miR-155 decreased the levels of the antioxidant proteins (Fig. 6). Thus, our results indicate that miR-155 mediated ATO resistance in A549R cells through the same cell signaling pathways as it mediates cellular resistance to other chemotherapeutic drugs. Our study further suggests that miR-155 plays a central role in mediating anticancer resistance through regulating the Nrf2 signaling pathway and can be developed as a new target for combating drug resistance of lung cancer.

Our results are consistent with previous reports showing that miR-155 plays an important role in anticancer drug resistance in cancer cells^19,21^. However, a recent study has shown that miR-155 can suppress the Nrf2 signaling pathway in responding to oxidative stress induced by a different chemical compound in a different type of cells. This was demonstrated by a role of miR-155 in mediating hepatocytotoxicity induced by an environmental toxicant, perfluorooctane sulfonate (PFOS) *in vitro* and *in vivo* ^27^. In contrast to its role in mediating ATO resistance in lung cancer cells, miR-155 significantly suppresses Nrf2 expression and activation in normal hepatocytes of rats in responding to PFOS-induced oxidative stress^27^. This indicates that miR-155 plays an opposite role in cancer cells verse its role in normal hepatocytes. This also suggests that miR-155 can function differently in different types of cells which adopt their unique cellular metabolism. The controversial results indicate that miR-155 plays a complicated role in mediating cellular responses to oxidative stress in cancer and normal cells. The different roles of miR-155 in regulating the Nrf2 signaling pathway in different types of cancer and normal cells need to be further elucidated in the future.

In this study, we explored the mechanisms underlying a high level of ATO resistance in A549R cells, which were established from A549 cells that were repeatedly exposed to increasing concentrations of ATO. We found that the Nrf2 level in A549R cells was significantly higher than that in A549 cells indicating that Nrf2 was upregulated, and this subsequently promoted cell survival, thereby conferring ATO resistance in A549R cells. The results are consistent with previous studies showing that upregulation of Nrf2 mediates drug resistance in cancer cells by promoting cell survival^13,28,29^. It should be noted that A549 cells bear a variety of mutations in the Keap1 gene, and this disrupts Keap1-Nrf2 interaction, thereby activating Nrf2 in the cells^30^. Here, we were able to detect sufficient difference in the Nrf2 level between A549R cells and A549 cells that exhibit constitutive activation of Nrf2 (Fig. 3). Thus, it is conceivable that the contribution of Nrf2 to ATO resistance in lung cancer cells would be more evident if this were compared to normal cells that exhibit a low level of Nrf2.

Our previous studies have shown that ATO induces cell apoptosis through ROS-mediated endoplasmic reticulum stress and mitochondrial dysfunction^14^. Since Bax and Bcl-2 can also induce cell apoptosis and mitochondrial dysfunction via endoplasmic reticulum stress^31^, it is possible that miR-155 may mediate ATO resistance in A549R cells through regulation of Bax/Bcl-2 ratio and apoptosis by modulating endoplasmic reticulum stress. Also it has been shown that miR-155 can downregulate the expression level of apoptotic protease activating factor 1 (Apaf-1), thereby inhibiting cell apoptosis induced by cisplatin in lung cancer cells^21^. Moreover, overexpression of miR-155 in lung cancer cells inhibits the expression level of forkhead box O3 (FOXO3a) protein that mediates cell apoptosis, decreasing the sensitivity of lung cancer cells to gefitinib^32^. In addition, miR-155 is actively involved in the chemotherapeutic drug resistance by downregulating a series of pro-apoptotic proteins that include tumor protein p53-inducible nuclear protein 1 (TP53INP1), X-linked inhibitor of apoptosis protein (XIAP), and suppressor of cytokine signaling 6 (SOCS6) among others^33,34^. Thus, it is likely that miR-155 may mediate ATO resistance in A549R cells by inhibiting cell apoptosis through downregulation of Apaf-1, FOXO3a, TP53INP1, XIAP, and SOCS6. The roles of miRNA-155 in regulating these target genes and mediating lung cancer chemotherapeutic drug resistance may be elucidated in the future.

In summary, in this study, we provide new insights into a role of miR-155 in mediating ATO resistance in lung cancer cells by activating the Nrf2 signaling pathway, increasing cellular antioxidant capacity and promoting cell survival through modulating cellular apoptotic process. Our results will facilitate the development of miR-155 as a new target for combating ATO resistance during lung cancer treatment.

## Methods

### Cell culture

Human lung adenocarcinoma A549 cell line was purchased from Gene Therapy Cancer Drug Engineering Research Center, Chengdu Huasun Group Inc., Ltd. (Chengdu, China). High glucose Dulbecco’s Modified Eagle’s Medium (DMEM, Gibco Life Technologies, Grand Island, NY, USA) supplement with 10% (v/v) fetal bovine serum, 100 U/ml penicillin and 100 μg/ml streptomycin was used for culturing cells. All cell cultures were routinely maintained in a humidified incubator with 95% air and 5% CO_2_ at 37 °C. Cells were subcultured to 80–90% confluence for the experiments.

### Establishment of the ATO resistant A549R cell line

The A549R cell line was established from the parental A549 cell line according to a method described previously with modifications^35,36^. Briefly, A549 cells were initially exposed to 5 µM ATO (YiDa Pharmaceutical Co. Ltd. Harbin Medical University, Heilongjiang, China) in complete DMEM, and subsequently exposed to the increasing doses of ATO by 2.5 µM every five passages. Cellular exposure to ATO continued for six months until cells were able to survive in medium with 20 µM ATO. The A549 cell line that exhibited resistance to 20 µM ATO was designated as the A549R cell line.

### Cell transfection

The miR-155 mimic, the miR-155 inhibitor, and the controls were purchased from Bioneer (Korea Bioneer Biotech Corp, Daejeon, South Korea). According to the manufacturer’s instructions, cells were seeded in 6-well plates at a density of 2 × 10^5^ per well overnight in media without antibiotics. Cells were then transfected with 40 pmol of the miR-155 mimic, the miR-155 inhibitor, and the controls with transfection reagent Lipofectamine 2000 (Invitrogen) in fresh medium without serum and antibiotics. Cells were cultured for 5 hours and subsequently were supplied with fresh medium with serum and cultured for additional 48 hours before they were harvested.

### Cell viability assay

Cell viability was determined by MTT [3-(4,5-dimethylthiazol-2-yl)-2,5-diphenyl-tetrazolium bromide] assay as described previously^14^. Briefly, A549R cells or A549 cells were seeded in 96-well plates at 1 × 10^4^ per well overnight. Cells were treated with different concentrations of ATO (0 to 150 µM for A549R cells and 0 to 30 µM for A549 cells) for 72 hours. Cells were then incubated with 100 µl of 0.5 mg/ml MTT at 37 °C for additional 4 hours in the dark. Subsequently, 100 µl of dimethylsulfoxide was added to dissolve formazan crystals. Absorbance at 570 nm (OD_570_) was measured with a microplate reader (Multiskan™GO, Thermo Fisher Scientific, Inc., Waltham, MA, USA). Cell viability was calculated using the equation: cell viability (%) = (OD_570_ of the treated group/OD_570_ of the control group) × 100%.

### Calculation of drug resistance index

The Drug Resistance Index (RI) was determined to evaluate the resistance of A549 cells to ATO according to a method described previously^35,36^. Initially, a 50% inhibitory concentration (IC_50_) of ATO was calculated with CompuSyn software (CompuSyn, Inc. Paramus, NJ 2007). Subsequently, RI value was calculated according to the equation: RI = IC_50_ of A549R cells/IC_50_ of A549 cells. A high RI value indicates a high level of cellular resistance to ATO.

### Determination of colony formation

Cells were seeded in 12-well plates at 1,000 cells/well, and cultured overnight and transfected with the miR-155 mimic, the miR-155 inhibitor, and the controls, respectively. Cells were then treated without or with 20 μM ATO for 24 hours. Subsequently, cells were washed with phosphate buffer solution (PBS) and supplied with fresh medium with serum. Cells were then cultured for additional 14 days allowing the formation of colonies. Colonies formed were washed with PBS, fixed in methanol, and stained with 10% (w/v) Giemsa. Colonies were counted under a microscope (Eclipse TiU, Nikon Corp., Tokyo, Japan), and their pictures were taken with a digital camera (Canon, Japan). The percentage of colony formation for each group was calculated with the equation: percentage of colony formation = a number of colony/1000 × 100%.

### Detection of cell migration

Cell migration was determined according to a previously reported method with modifications^37^ with a 24-well invasion chamber containing an upper and lower chamber with an 8 μm pore in between (Corning Life Science, Acton, MA). Briefly, cells were harvested and resuspended in DMEM containing 1% (v/v) fetal bovine serum. Subsequently, 100 μl of cell suspension (1 × 10^5^ cells in total) was added into the upper chamber while the lower chamber was filled with 600 μl of DMEM containing 10% (v/v) fetal bovine serum without or with 20 μM ATO. After 24 hours of incubation, adherent cells in the lower chamber were fixed with methanol and stained with 10% (w/v) Giemsa. The pictures of five randomly chosen areas were captured with a microscope at 100× magnification for quantification analysis.

### Determination of cellular distribution of Nrf2 by immunofluorescence

Immunofluorescence was conducted to determine the distribution of Nrf2 in the nucleus and cytoplasm according to a method described previously^14^. In brief, 5 × 10^5^cells were seeded in a 6-well plate, which contained a sterilized slide (24 mm × 24 mm) at the bottom of wells. After ATO treatment, cells were fixed with 4% para-formaldehyde for 10 min, incubated with 5% bovine serum albumin for 1 hour at room temperature, and washed with PBS for three times. Subsequently, cells were incubated with a rabbit anti-Nrf2 polyclonal antibody (KeyGen Bio-technology, Nanjing, China, 1:100) at 4 °C overnight followed by incubation with a Cy3-conjugated secondary antibody for additional 1 h at 37 °C. Cells were washed for three times and then incubated with 4′,6-diamidino-2-phenylindole (DAPI) at room temperature for 10 min. All slides were sealed with an anti-fluorescence quenching agent and then examined under a fluorescent microscope (Eclipse TiU, Nikon Corp., Tokyo, Japan) at 400× magnification. Nrf2 and nuclei exhibited red and blue fluorescence, respectively.

### Determination of cellular protein levels by immunoblotting

The whole cell extracts were made with cell lysis buffer (Beyotime Institute of Biotechnology, Shanghai, China). Nuclear and cytosolic extracts of cells were made with the Nuclear Extraction Kit from KeyGEN (KeyGEN Biotechnology, Nanjing, China) according to the manufacturer’s instructions. Protein concentrations were determined with the BCA Protein Assay Kit (ComWin Biotech Co., Ltd. Beijing, China). Fifty microgram proteins were subjected to 12% SDS-PAGE gel electrophoresis and transferred onto a polyvinylidene difluoride (PVDF) membrane. The membrane was blocked in 5% non-fat milk for 2 hours at room temperature. Proteins were probed with primary antibodies against Nrf2, Lamin B, HO-1 and NQO1 (Boster Biological Technology, Wuhan, China, 1:500), Bcl-2, Bax (KeyGen Bio-technology, Nanjing, China, 1:200) and β-actin (ZSGB Bio, Beijing, China, 1:1000), respectively. The membrane was incubated with a secondary antibody conjugated with horseradish peroxidase (ZSGB Bio, Beijing, China, 1:6000) for 1 hour at room temperature. Proteins were detected by an enhanced chemiluminescent reagent and visualized by the Molecular Imager Gel Doc XR System (Bio-Rad, Hercules, CA, USA). The intensity of the protein bands was quantified with the QuantityOne Image Software (Bio-Rad, Hercules, CA, USA). Lamin B and β-actin were used as the internal control. The levels of protein expression were calculated relative to the level of Lamin B or β-actin. “Fold change in protein expression” was calculated based on the protein level in the control cells.

### Detection of apoptosis

Cellular apoptosis was measured with an Annexin V-fluorescein isothiocyanate (Annexin V-FITC) and propidium iodide (PI) dual staining detection kit (KeyGen Bio-technology, Nanjing, China) according to the manufacturer’s instructions. Briefly, cells (2 × 10^5^ cells/well) were seeded in 6-well plates and cultured overnight. Cells were treated without or with 20 µM ATO for 24 hours. Floating and attached cells were harvested and subsequently washed twice with cold PBS. Cells were resuspended in 500 μl of binding buffer and stained with 5 μl of PI and 5 μl of Annexin V-FITC at room temperature for 30 min in the dark. Cells (20,000 cells per sample) were immediately analyzed by flow cytometry (Beckman coulter, FC500, FL, USA).

### Determination of cellular expression of miR-155 by real-time quantitative PCR

Total RNAs (<200 nt) were extracted with a miRcute miRNA Isolation Kit according to the manufacturer’s protocol (Tiangen Biotech, Beijing Co., Ltd). Total RNAs were then transcribed into cDNA with a miRcute miRNA First-Strand cDNA Synthesis Kit (Tiangen Biotech, Beijing Co., Ltd). Subsequent PCR reactions were assembled in a 20 µl system with a miRcute miRNA qPCR Detection Kit (Tiangen Biotech, Beijing Co., Ltd) with a miR-155 specific forward primer. The forward primers of miR-155 and hsa-U6 were purchased from Tiangen Biotech, and the reverse primer was provided in the miRcute miRNA qPCR Detection Kit (Tiangen Biotech, Beijing Co., Ltd). PCR amplification was conducted according to the conditions: 1 cycle of initial denaturation (95 °C for 2 min), 40 cycles of amplification (94 °C for 20 s and 60 °C for 34 s). Quantitative real-time PCR was performed on a thermal cycler from Bio-Rad Laboratories. Results were analyzed with ABI SDS version 2.3. The relative expression level of miR-155 was calculated using the 2^−∆∆Ct^ method. Briefly, the cycle threshold (Ct) values were initially normalized to hsa-U6 in the same sample and designated as ∆Ct values. Subsequently, ∆∆Ct values were obtained by subtracting the ∆Ct values of the control samples from those of the treated samples.

### Statistical analysis

All results were obtained from at least three independent experiments and were presented as means ± standard deviation (SD). Statistical differences were analyzed and detected with the Statistical Program for Social Sciences, version 17.0 (SPSS Inc., Chicago, IL, USA). Statistical analysis was performed with an independent sample t-test and one-way analysis of variance (ANOVA). Student-Newman-Keuls (SNK) test was employed to compare the data of two independent groups. Non-parametric Kruskal-Wallis test was used when the original data bear variance heterogeneity. Tukey’s test was employed to determine the differences in the data of two independent groups. *P* < 0.05 denotes a statistical significance.
